# Fire legacies in eastern ponderosa pine forests

**DOI:** 10.1002/ece3.4879

**Published:** 2019-01-16

**Authors:** Caleb P. Roberts, Victoria M. Donovan, Carissa L. Wonkka, Larkin A. Powell, Craig R. Allen, David G. Angeler, David A. Wedin, Dirac Twidwell

**Affiliations:** ^1^ Department of Agronomy & Horticulture University of Nebraska Lincoln Nebraska; ^2^ School of Natural Resources Nebraska Cooperative Fish and Wildlife Research Unit Lincoln Nebraska; ^3^ School of Natural Resources University of Nebraska Lincoln Nebraska; ^4^ U.S. Geological Survey, Nebraska Cooperative Fish & Wildlife Research Unit, School of Natural Resources University of Nebraska Lincoln Nebraska; ^5^ Department of Aquatic Sciences and Assessment Swedish University of Agriculture Sciences Uppsala Sweden

**Keywords:** birds, burn severity, disturbance legacies, ecological memory, fire legacies, material legacies, ponderosa pine, stand structure

## Abstract

Disturbance legacies structure communities and ecological memory, but due to increasing changes in disturbance regimes, it is becoming more difficult to characterize disturbance legacies or determine how long they persist. We sought to quantify the characteristics and persistence of material legacies (e.g., biotic residuals of disturbance) that arise from variation in fire severity in an eastern ponderosa pine forest in North America. We compared forest stand structure and understory woody plant and bird community composition and species richness across unburned, low‐, moderate‐, and high‐severity burn patches in a 27‐year‐old mixed‐severity wildfire that had received minimal post‐fire management. We identified distinct tree densities (high: 14.3 ± 7.4 trees per ha, moderate: 22.3 ± 12.6, low: 135.3 ± 57.1, unburned: 907.9 ± 246.2) and coarse woody debris cover (high: 8.5 ± 1.6% cover per 30 m transect, moderate: 4.3 ± 0.7, low: 2.3 ± 0.6, unburned: 1.0 ± 0.4) among burn severities. Understory woody plant communities differed between high‐severity patches, moderate‐ and low‐severity patches, and unburned patches (all *p* < 0.05). Bird communities differed between high‐ and moderate‐severity patches, low‐severity patches, and unburned patches (all *p* < 0.05). Bird species richness varied across burn severities: low‐severity patches had the highest (5.29 ± 1.44) and high‐severity patches had the lowest (2.87 ± 0.72). Understory woody plant richness was highest in unburned (5.93 ± 1.10) and high‐severity (5.07 ± 1.17) patches, and it was lower in moderate‐ (3.43 ± 1.17) and low‐severity (3.43 ± 1.06) patches. We show material fire legacies persisted decades after the mixed‐severity wildfire in eastern ponderosa forest, fostering distinct structures, communities, and species in burned versus unburned patches and across fire severities. At a patch scale, eastern and western ponderosa system responses to mixed‐severity fires were consistent.

## 1 INTRODUCTION

Globally, changes are propagating in the timing, frequency, intensity, and attendant legacies of disturbances that lead to unique assortments of plant and animal species in many ecosystems (Turner, 2010; Vitousek, Mooney, Lubchenco, & Melillo, 1997). Disturbance legacies are defined as “biologically derived legacies that persist in an ecosystem or landscape following disturbance” (Cuddington, 2011; Johnstone et al., 2016). There are two types of disturbance legacies: information legacies, which are adaptations to a disturbance regime represented by the distribution of species traits in a community, and material legacies, which are the “biotic and abiotic residuals” (e.g., post‐disturbance structures and community compositions) that remain in an ecosystem following a disturbance event (Johnstone et al., 2016). Material legacies influence the trajectory of post‐disturbance systems because they provide individuals that subsequently make up the community and the physical materials that influence the establishment of individuals in an area (Franklin & MacMahon, 2000; Johnstone, Hollingsworth, Chapin, & Mack, 2010; Peterson, 2002). Material legacies thereby provide ecological memory of the pre‐disturbance system to recovering systems, making them instrumental in keeping systems within “safe operating spaces” (Johnstone et al., 2016; Peterson, 2002). Because material legacies are determined by the particular characteristics of a disturbance, alteration of disturbance regimes often involves alteration of material legacies (e.g., Collins, Stephens, Moghaddas, & Battles, 2010; Tinker & Knight, 2000). Over time, altering material legacies can lead to an erosion of ecological memory as material legacies are lost or altered (Johnstone et al., 2016). However, in many cases, it is unclear how long material legacies persist and remain important influences of biotic structures and communities post‐disturbance.

Fire is one of the most altered disturbance regimes on the planet (Bowman et al., 2009; Keane et al., 2002; Twidwell et al., 2016). Human actions that reduce fire severity, frequency, and distribution can unintentionally eliminate material legacies that keep systems within “safe operating spaces” (Carpenter, Brocks, Folke, Nees, & Scheffer, 2015; Dale et al., 2001). For instance, current forest management policies often attempt to constrain fire regimes to low severity, suppress fire altogether, or mitigate effects of severe and mixed‐severity fires via thinning treatments or post‐fire salvage logging (Covington et al., 1997; Reynolds et al., 2013). Efforts are growing to disentangle past fire legacies from contemporary trajectories in order to determine their role in shaping historical ecosystem structure and composition and maintaining safe operating spaces (Metlen, Skinner, Olson, Nichols, & Borgias, 2018; Odion et al., 2014; Swetnam et al., 2016). But as fire regime alteration becomes more prevalent in forested systems, opportunities to study material legacies of fire over longer time scales have become increasingly rare (Hutto et al., 2016), limiting our understanding of how long material legacies persist following disturbance (Odion & Hanson, 2013).

**Figure 1:**
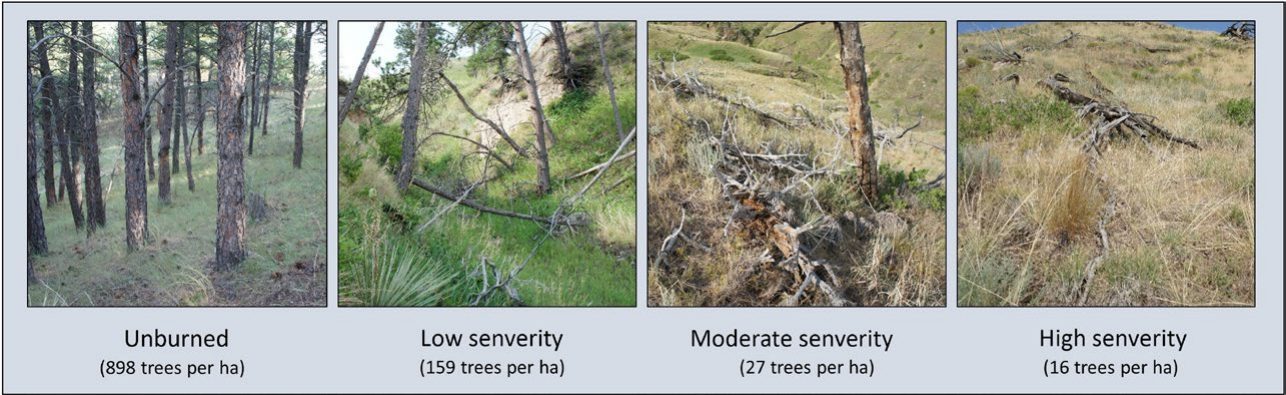
Images of a typical study site sampled in the summer of 2016 for unburned forest and low‐, moderate‐, and high‐severity burned forests from the 1989 Fort Robinson wildfire

Ponderosa pine (*Pinus ponderosa*) forests of North America are fire‐dependent systems thought to require only frequent, low‐intensity fire to retain safe operating space and biodiversity (Brown, Agee, & Franklin, 2004; Scholl & Taylor, 2010). Recent studies have questioned these assumptions: they suggest that historically, ponderosa systems also experienced mixed‐severity fires, defined by variability in intensities (including some areas of high intensity), every several decades or centuries (Odion et al., 2014; Williams & Baker, 2012). These mixed‐severity fires are thought to have led to diversity in forest succession and stand structure across the burned area (Williams & Baker, 2012; Figure 1). Further studies have debated the importance of mixed‐severity fire in maintaining ponderosa systems (Fulé et al., 2014; Levine et al., 2017), particularly its historic frequency (Merschel, Heyerdahl, Spies, & Loehman, 2018) and geographic ubiquity (Stevens et al., 2016). Because ponderosa systems vary across their geographic range, it is unclear that either view of the role of mixed‐severity fire regimes holds across the entire North American continent (Fulé et al., 2014; Odion et al., 2014). For instance, the relatively contiguous (at a landscape scale) western ponderosa forests embedded in mixed‐conifer systems may respond differently to mixed‐severity fire than eastern ponderosa pine systems (i.e., within the Great Plains of North America) that are typified by an ecotonal, patchy spatial distribution of ponderosa monocultures within grassland matrices at landscape scales (Brown & Sieg, 1999).

Despite the debate on its historic prevalence, examples exist of mixed‐severity fire and resultant legacies promoting diversity in ponderosa pine systems (DellaSala et al., 2017; Figure 1). For example, the diversity of biotic structures (e.g., varying snag, live tree, and coarse woody debris) resulting from mixed‐severity fire in ponderosa pine systems fosters diversity in biotic communities (Huffman, Meador, Stoddard, Crouse, & Roccaforte, 2017; Malone et al., 2018). Numerous species require the open habitats created by high‐severity fire (e.g., Hutto, 2008; Fornwalt & Kaufmann, 2014), others prefer low tree densities that low‐severity fire fosters (Abella & Fornwalt, 2015; Kotliar, Kennedy, & Ferree, 2007), and still other species require high tree densities retained in areas that escape fire (Fontaine & Kennedy, 2012). Additionally, there is evidence that mixed‐severity fire legacies can provide ponderosa systems with adaptations to future environmental changes such as climate change and the resultant disturbance regimes (e.g., more frequent droughts and fires; Baker, 2018).

Determining how mixed‐severity fire material legacies affect and maintain diversity in ponderosa pine systems will require studying systems in which disturbance legacies remain unaltered, an increasingly difficult task due to pervasive human alterations of legacies (Donato et al., 2006; Hutto & Patterson, 2016). Additionally, quantifying the role of mixed‐severity fires in structuring ponderosa systems where their prevalence is unknown but are nevertheless considered “catastrophic threats”, such as in eastern ponderosa pine systems (Schneider, Humpert, Stoner, & Steinauer, 2005), provides data‐driven assessment of the impacts of mixed‐severity fire on shaping ecosystems. Here, we aim to quantify the characteristics and persistence of material legacies that arise from variation in fire severity in an eastern ponderosa pine forest. We quantified biotic residuals of disturbance (one aspect of material legacies) by measuring forest stand structure (tree density and coarse woody debris) and biotic communities (understory woody plant and bird communities) within a 27‐year‐old mixed‐severity wildfire perimeter that experienced minimal pre‐ or post‐fire management treatment. This provides a rare example of relatively unaltered material legacies three decades after disturbance.

## 2 MATERIALS AND METHODS

### 2.1 Study site

We conducted this study in the Pine Ridge region of Nebraska, USA, in 2016. The Pine Ridge Escarpment is a semiarid region in the northwestern corner of Nebraska marking the northern border of the Northern High Plains and the southern border of the unglaciated Missouri Plateau (Urbatsch & Eddy, 1973). The escarpment sits hundreds of meters above the surrounding plains and is characterized by rocky ridges, vertical slopes, and deep canyons with a mean elevation of approximately 1,219 m. The escarpment is an ecotonal region characterized by ponderosa pine interspersed with mixed grass prairie (Schneider et al., 2005). Being ecotonal, the Pine Ridge hosts both forest (e.g., *Mahonia repens*, *Prunus virginiana*) and grassland species (e.g., *Artemesia tridentata*, *Ericameria* sp.; Johnsgard, 2005). Likewise, both eastern (e.g., Eastern Kingbird, Eastern Bluebird) and western (e.g., Western Kingbird, Mountain Bluebird) North American species inhabit the escarpment (Johnsgard, 2005).

**Figure 2:**
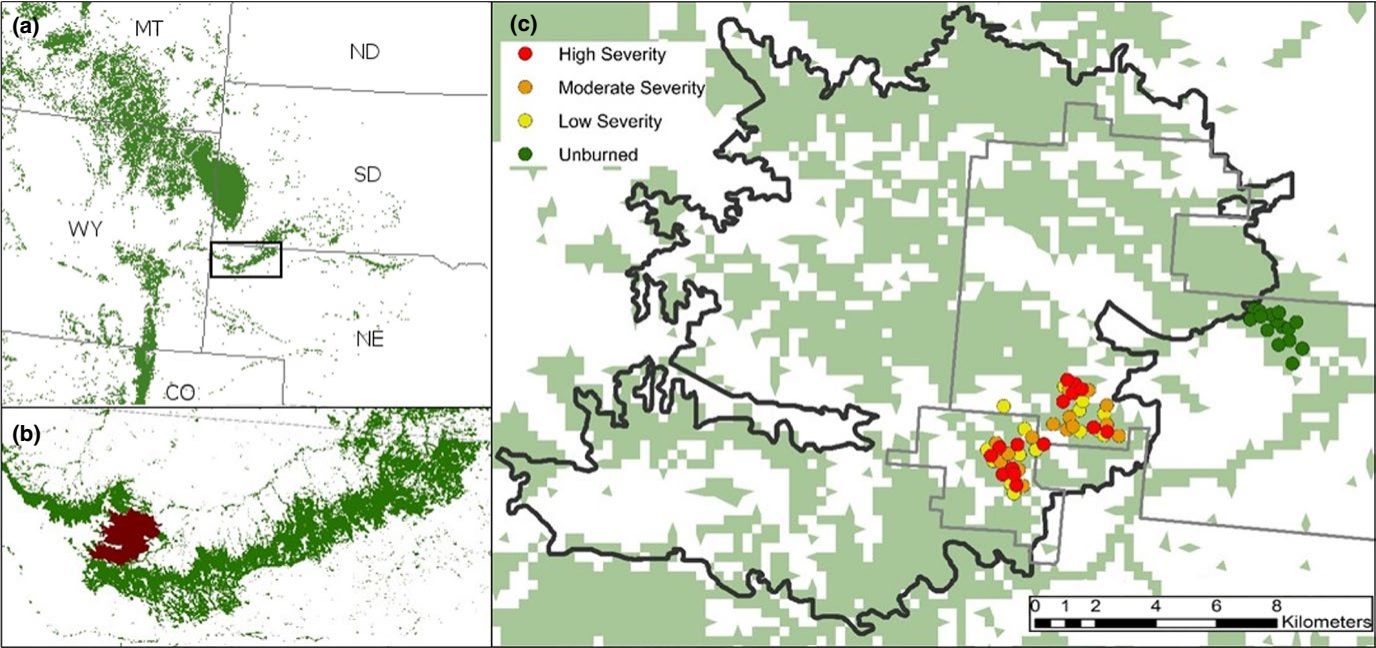
(a) The northeastern distribution of ponderosa pine in the United States provided by the US Forest Service indicated in green. (b) The Pine Ridge region of Nebraska, with the 1989 Fort Robinson wildfire in red and ponderosa pine distribution in green. (c) The distribution of sampling sites of different fire severity classes, indicated by colored points, within the Fort Robinson (42.6693°N, 103.4689°W)wildfire perimeter (black), ponderosa distribution in green and public land boundaries for Fort Robinson State Park and Peterson Wildlife Management Area (gray).

Although the Pine Ridge is largely thought to have experienced a low‐severity wildfire regime (Brown & Sieg, 1999; Savage & Mast, 2005), the region has experienced multiple large mixed‐severity fires over at least the last three decades (MTBS, 2016). In 1989, the Fort Robinson mixed‐severity wildfire burned 18,975 ha across the Pine Ridge escarpment, much of which occurred within Fort Robinson State Park and the Peterson Wildlife Management Area (42.6693°N, 103.4689°W; Figure 2). We define mixed‐severity fire following Agee's (1990, 1993) definition, where 20% to 70% of the fire that occurred in forested areas was stand replacing. Approximately 1,330 ha were classified as high‐severity, 3,604 ha as moderate‐severity, and 5,971 ha as low‐severity within the fire perimeter. Areas that were designated as moderate and high severity were limited to forested regions, while low‐severity areas burned through both forest and grasslands. Prior to the 1989 fire, land management suppressed all fire. However, for the past 27‐year post‐fire, the burned area has received no post‐fire treatments or manipulations such as salvage logging or tree thinning. Limited cattle (*Bos taurus*) and horse (*Equus caballus*) grazing has occurred across the study area pre‐ and post‐fire.

### 2.2 Site selection

In summer 2016, we collected data at Fort Robinson State Park and the Peterson Wildlife Management Area, two public lands within the Pine Ridge Escarpment that burned in the Fort Robinson wildfire of 1989. We selected 3,598 ha with adequate road access within these public lands that captured a full range of burn severities and unburned forest (Figure 2). Unburned and burned forest sites occurred on the same butte system, separated by a ~4 km pasture area. Burn severity classes followed the Monitoring Trends in Burn Severity project (MTBS, 2016) severity designations (unburned forest, low‐severity burned forest, moderate‐severity burned forest, high‐severity burned forest; Eidenshink et al., 2007).

We used a stratified‐random design to distribute 14 sampling sites in each of the four burn severity classes for a total of 56 sites. We mapped burn severity classes to the study area with MTBS geospatial raster data for the 1989 Fort Robinson fire. For each burn severity class, we randomly generated 14 points and selected the closest patch of that class to each randomly generated point. We then placed sites in a central location within the patch. Because both grassland and forest are categorized under the same burn severity classes in MTBS data, we used historic USGS satellite imagery from Google Earth to confirm that forest, rather than grassland, was present at the time of the wildfire for each burn class. We separated all sites by a minimum of 100 m (Buckland et al., 2001).

To identify legacy effects 27 years after a mixed‐severity fire, we collected forest stand structure, understory woody plant community, and bird community data at each sampling site. For stand structure, we surveyed tree density and total coarse woody debris cover. For community data, we estimated understory woody plant and bird community compositions and richness.

### 2.3 Tree density and coarse woody debris

We used the point‐center quarter method to estimate tree density, placing a single point at each sample site (Cottam & Curtis, 1956). At each site, we estimated live tree and snag densities. We defined live trees as woody plants standing ≥1.4 m (diameter at breast height). Similarly, we defined snags as free‐standing dead trees ≥1.4 m in height. Because of tree scarcity across many of our sites, we only measured trees up to 100 m from the point center. If no trees were within this distance, we entered a value of 101 m.

To sample coarse woody debris (woody debris with a diameter greater than or equal to 10 cm; CWD), we established a 30‐m transect in a randomly selected north–south or east–west direction at each site. We measured the length of the transect line that was covered with CWD and then divided this value by the total transect length (30 m), multiplied by 100, to determine the percent CWD cover at each site.

### 2.4 Understory woody plant community composition

To estimate understory woody plant community composition, we collected species presence–absence data at each sampling site. We defined understory woody plants as woody plants <1.4 m in height (i.e., less than diameter at breast height level). We distributed five circular sampling plots with 5‐m radii around each sampling site. We placed one at the center of the sampling site, and the four others 15 m from center of the sampling site in each of the cardinal directions. In each plot, we recorded all understory woody plant species rooted within the plot. If a species was present in any of the five plots, we counted it as present for the sampling site.

### 2.5 Bird community composition

From May 25 to June 6, we estimated bird community composition from species presence–absence data. At each sampling site, we recorded bird species presence with visual and acoustic point‐count surveys. We conducted surveys within a 5.5 hr sampling window starting 30 min prior to sunrise and ending five hours after sunrise. We did not survey if winds exceeded 20 km/hr or during precipitation events (Flanders et al, 2006; Huff, Bettinger, Ferguson, Brown, & Altman, 2000). For each point‐count survey, we recorded all bird species we saw or heard during a five‐minute period within 50 m of the point to ensure recorded species were using the burn severity at which the point was situated and to maximize detection probability (Buckland et al, 2001). We revisited each point once within 5 days to increase the probability of capturing all present species (Sliwinski, Powell, Koper, Giovanni, & Schacht, 2015). For analyses, we pooled the species recorded from both visits.

### 2.6 Analyses

We used general linear models to test for legacy effects of burn severity on tree density and CWD cover. To differentiate legacy effects on patterns in live trees and snags, we developed separate models for live tree and snag density data. Where necessary, data were log‐transformed to meet model assumptions. We conducted multiple comparisons of slopes among burn severities with false discovery rate *p*‐value adjustments (Hothorn, Bretz, & Westfall, 2008; “glht” function; R package multcomp).

To examine differences in understory woody plant and bird community composition across burn severities, (a) we estimated mean species richness by severity class and compared 95% confidence limits across severities and (b) we compared multivariate community composition by severity class. To compare multivariate community composition, we first assessed community compositions visually via ordination. Because our data were presence–absence and fit unimodal assumptions (i.e., we sampled across the full range of burn severities), we use canonical correspondence analysis (CCA), setting burn severity as the constraining variable (Palmer, 1993). We estimated the mean center and 95% confidence limits for the site ordination scores of each burn severity category for the first two CCA axes.

Following ordination, we used a permutational multivariate analysis of variance (PERMANOVA) to confirm any significant community composition differences across burn severities (Anderson, 2005). We determined whether the overall effect of burn severity significantly predicted community composition. We then compared the community compositions of each burn severity, using false discovery rate p‐value adjustments for multiple comparisons. Because PERMANOVA can be sensitive to variability between groups, we tested for homogeneity of variances for all comparisons using the permutational test of multivariate dispersions (PERMDISP; Anderson & Walsh, 2013). All ordination and significance tests were conducted with R software using the “vegan” package (Oksanen et al., 2007; R Core Team, 2015).

## 3 RESULTS

### 3.1 Tree density

**Figure 3:**
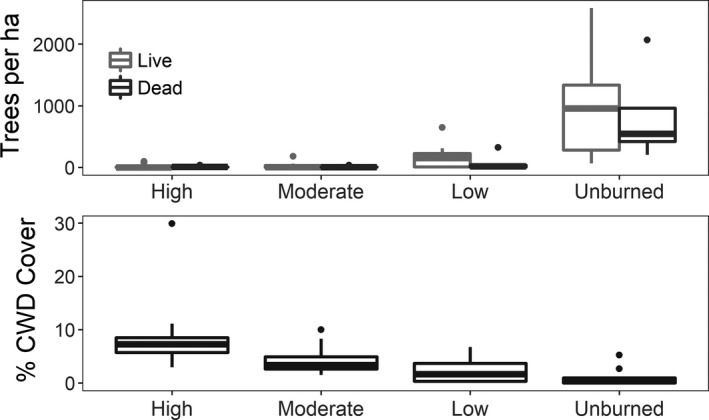
Forest stand structure across a 27‐year‐old burn severity gradient at Ft. Robinson State Park, Nebraska, 2016. The top panel shows boxplots of tree density per hectare for each burn severity class. The bottom panel shows boxplots of percent cover per 30 m transect of coarse woody debris for each burn severity class. The burn severity classes represent high‐severity (High), moderate‐severity (Moderate), low‐severity (Low), and unburned (Unburned)

Burn severity was a significant predictor of live (*F*_3,51_ = 26.260, *p* < 0.001) and dead (*F*_3,33_ = 18.250, *p* < 0.001; Supporting Information Table S1; Figure 3) tree density 27 years after fire (Figure 1). The live tree density generalized linear model (*y* = 1.221 + 2.646 (low) + 0.816 (moderate) + 5.184 (unburned)) indicated that low‐severity burn and unburned classes were positively related to tree density (*p* ≤ 0.001; Supporting Information Table S1). Only the unburned class was positively related to snag density in the dead density model (*y* = 2.029 + 0.684 (low) − 0.363 (moderate) + 4.357 (unburned); *p* < 0.001; Supporting Information Table S1; Figure 1). Live tree density differed significantly among all burn severity levels, except moderate and high severities (*t* = 0.816, *p* = 0.597 Supporting Information Table S1). Snag density only distinguished unburned patches from burned patches (all *p* < 0.001; Supporting Information Table S1).

### 3.2 Coarse woody debris

Coarse woody debris varied across the burn severity gradient 27 years after fire, with fire severity being a significant predictor of coarse woody debris cover (*F*_3,56_ = 16.74, *p* < 0.001; Figure 3; Supporting Information Table S1). Our coarse woody debris model indicated that moderate and high severities had similar coarse woody debris cover and low and unburned severities had similar coarse woody debris cover. Coarse woody debris was significantly higher in moderate‐ and high‐severity burned forest than in low‐severity and unburned forest (*y *= [8.479 − 6.149 (low) − 4.138 (moderate) − 7.526 (unburned)]^4^; Supporting Information Table S1).

### 3.3 Understory woody plant community composition

**Figure 4:**
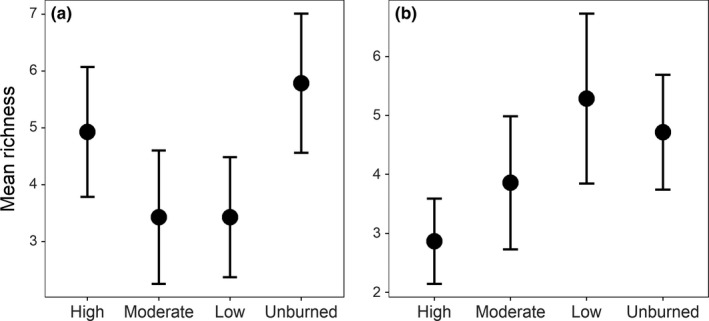
Mean understory woody plant (panel A) and bird (panel B) species richness by burn severity in Fort Robinson State Park, Nebraska, 2016. Bars indicate 95% confidence limits. The burn severity classes represent high‐severity (High), moderate‐severity (Moderate), and low‐severity (Low), and unburned (Unburned)

We observed 18 understory woody plant species across all sampling sites (Supporting Information Table S2), with 10 species in high‐severity patches, 12 in moderate‐severity patches, 9 in low‐severity patches, and 15 in unburned patches (Supporting Information Table S2). Moderate‐severity, low‐severity, and unburned patches had two, one and three unique species, respectively (Supporting Information Table S2). We observed no species unique to high‐severity patches (Supporting Information Table S2). Understory woody plant richness was highest in unburned (5.93 ± 1.10) and high‐severity (5.07 ± 1.17) patches, and it was lower in moderate (3.43 ± 1.17) and low‐severity patches (3.43 ± 1.06; Figure 4).

**Figure 5:**
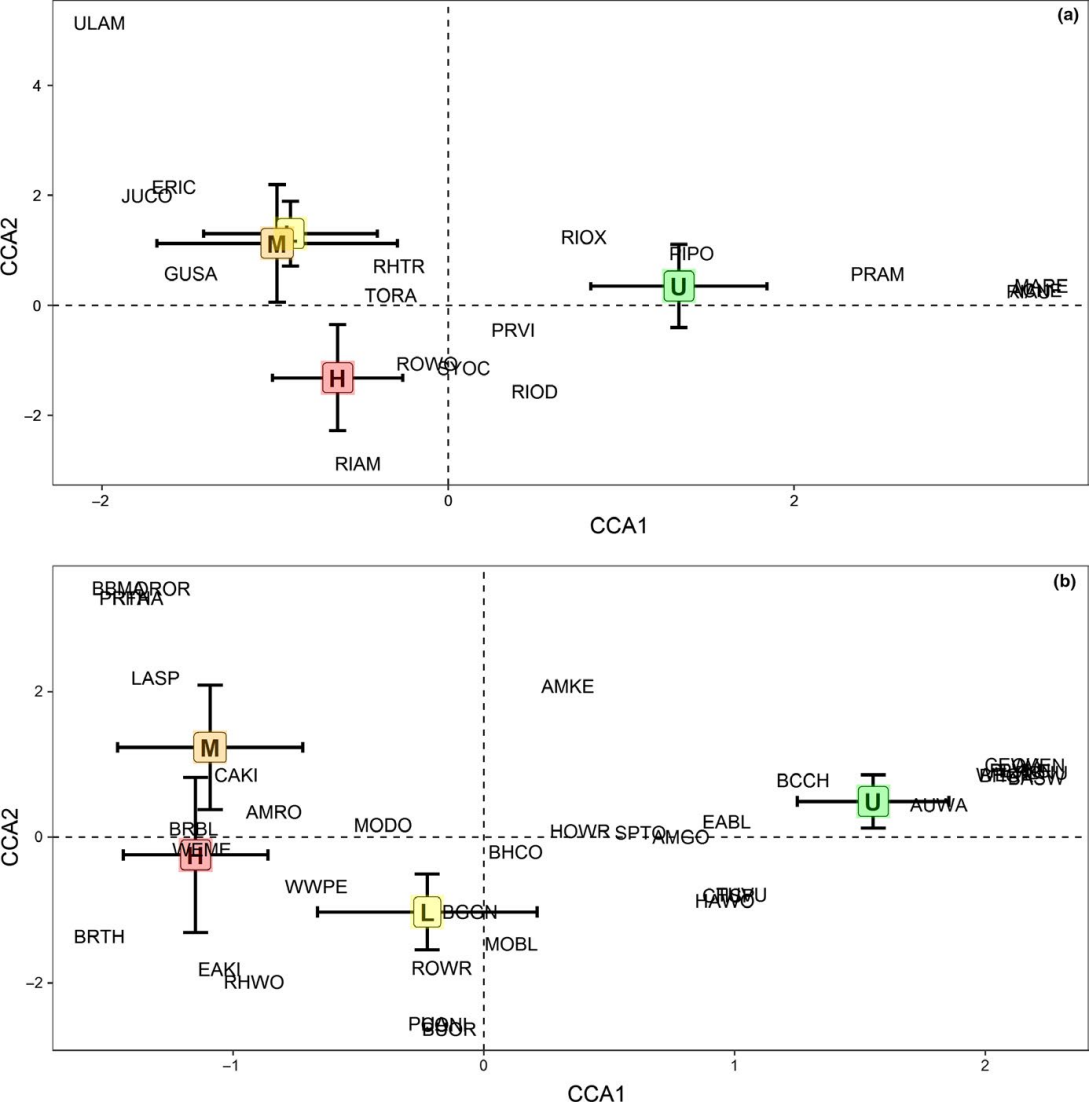
Mean constrained site scores and species scores for the first two axes of a canonical correspondence analysis for understory woody plant (panel A) and bird (panel B) community composition data across a burn severity gradient in Fort Robinson State Park, Nebraska. Bars indicate 95% confidence limits of the mean site scores. The burn severity classes represent high‐severity (H), moderate‐severity (M), low‐severity (L), and unburned (U). Plant species scores are represented by the first two letters of genus and the first two letters of species, and bird species scores correspond to American Ornithological Union species abbreviations. See Supporting Information Table S2 and S4 for plant and bird species, respectively

In the CCA, the constraints explained 11.8% of variance, and the three constrained axes explained 59.6%, 22.9%, and 17.5% of total constrained variance, respectively. Because the first two CCA axes explained approximately 83% of the total constrained variance, we only considered these axes in the results. The first CCA axis distinguished burned and unburned communities, and the second CCA axis differentiated high‐severity communities from moderate and low‐severity communities (Figure 5). The mean and 95% confidence limits of the constrained site scores from the first and second CCA axes and PERMANOVA results confirmed that burn severity was a significant predictor of understory woody plant community composition 27 years after wildfires (pseudo *F*_3,53_ = 4.502, *p* = 0.001; Figure 5; Supporting Information Table S3). Multiple PERMANOVA comparisons corroborated the CCA results as well: both unburned and high‐severity understory woody plant communities were distinct from all others (all *F* > 9.099, all *p* = 0.002; Supporting Information Table S3). Low‐ and moderate‐severity communities were not different (*F* = 0.663, *p* = 0.624; Supporting Information Table S3). High‐severity communities differed from all others (all *F* > 5.680, all *p* < 0.005; Supporting Information Table S3). Ordination showed that *Pinus ponderosa* strongly associated with unburned sites; additionally, two *Ribes* species (*Ribes oxyacanthoides* and *Ribes aurem*), *Prunus americana*, *Prunus virginiana*, *Ribes odoratum,* and some mesophilic plants such as *Mahonia repens* and *Acer negundo* associated with unburned sites (Figure 5). *Prunus virginiana* and *Ribes odoratum* were also associated with high‐severity sites; *Rosa woodsii*, *Symphoricarpos occidentalis*, and *Ribes americanum* were also common in high‐severity sites (Figure 5). Moderate‐ and low‐severity communities showed high overlap and shared several species including *Ulmus americana*, *Juniperus communis*, *Ericameria *sp., *Gutierrezia sarothrae*, *Rhus trilobata*, and *Toxicodendron radicans *(Figure 5).

### 3.4 Bird community composition

We observed 40 bird species throughout the study area (Supporting Information Table S4). We observed a total of 14 species in high‐severity, 19 in moderate‐severity, 24 in low‐severity, and 23 in unburned classes (Supporting Information Table S4). We also detected unique bird species in each burn severity category that were not found in other areas: 1 in high‐severity, 3 in moderate‐severity, 2 in low‐severity, and 8 in unburned (Supporting Information Table S4). Bird species richness patterns differed from understory woody plant richness patterns (Figure 4). Bird species richness varied across burn severities: low‐severity patches had the highest (5.29 ± 1.44) and high‐severity patches had the lowest (2.87 ± 0.72; Figure 4).

Like understory woody plant community composition, in the bird CCA, constraints explained 10.1% of the variance, and the three constrained axes explained 59.3%, 24.5%, and 16.3% of the total constrained variance. Again, the first and second CCA axis site scores differentiated three bird communities (explaining approximately 84% of constrained variance), based on means and 95% confidence limits (Figure 5). But in this case, the first CCA axis differentiated both burned versus unburned as well as burn severities; whereas the second axis only slightly distinguished burn severities (Figure 5). Regardless, the initial PERMANOVA also confirmed CCA results, demonstrating that burn severity was a significant predictor of bird community composition (pseudo *F*_3,53_ = 6.193, *p* = 0.001; Supporting Information Table S3), and the PERMDISP did not find heterogeneity in spread (pseudo *F*_3,53_ = 0.840, *p* = 0.479). Multiple PERMANOVA comparisons reiterated differences shown in the CCA (Supporting Information Table S3). Bird communities did not differ between high‐ and moderate‐severities (*F* = 1.284, *p* = 0.260), but bird communities in low‐severity and unburned patches differed from all other patch types (Supporting Information Table S3). Unburned sites were strongly characterized by several species typical of closed‐canopy habitats such as Yellow‐rumped Warbler, Black‐capped Chickadee, Cedar Waxwing, and Plumbeous Vireo, and unburned sites were also loosely associated with some cavity‐nesting species such as American Kestrel, Eastern Bluebird, and House Wren (Figure 5). Low‐severity sites shared species with unburned sites such as House Wren, Spotted Towhee, American Goldfinch, and Chipping Sparrow (Figure 5), and low severity also shared some species with high‐/moderate‐severity sites, including several open habitat‐associated species such as Western Meadowlark, Cassin's Kingbird, and Rock Wren, as well as some late decay stage cavity nesters such as Red‐headed Woodpeckers and Northern Flickers (Figure 5). High‐ and moderate‐severity sites were strongly associated with open habitat‐associated species such as Lark Sparrow and Western Meadowlark (Figure 5).

## 4 DISCUSSION

Mixed‐severity fire created multidecadal material legacies in forest stand structure and biotic communities in an eastern ponderosa pine forest. We identified distinct tree densities, coarse woody debris cover, understory woody plant communities, and bird communities across the current landscape which coincide with a burn severity gradient from a fire that occurred 27 years prior to sampling. Stand structure, understory woody plant communities, and bird communities differed between unburned, high‐severity, and low‐severity burn patches. Even 27‐year post‐fire, low‐severity burn patches hosted many grassland bird species (e.g., Western Meadowlark, Cassin's Kingbird) that were absent in unburned forest patches while also maintaining relatively high tree densities. High‐severity burn patches still tended strongly toward grassland conditions, with low tree density and understory woody plant and bird species typical of grasslands. Moderate‐severity burn patches showed the least distinction, overlapping with high severity in tree density and bird communities and with low severity in coarse woody debris and understory woody plant communities. Although we detected the highest number of unique species and species richness in unburned patches of ponderosa, we also detected unique structures, understory woody plant species, and bird species across a range of burn severities; further, we show that high‐severity patches supported higher understory woody plant species richness than moderate‐ or low‐severity patches 27‐year post‐fire. Thus, our study is among the first to show that mixed‐severity fire produces multidecadal material legacies that support unique species assemblages in eastern ponderosa pine systems. This contrasts with the assumption that mixed‐severity fire represents a “catastrophic” stressor for eastern ponderosa systems (Schneider et al., 2005). Our results also build upon shorter‐term studies in conifer systems demonstrating how fire legacies influence structure in burned versus unburned forests (Fontaine, Donato, Robinson, Law, & Kauffman, 2009; Hutto, 1995) and across fire severities (Fontaine & Kennedy, 2012; Stephens, Ausprey, Seavy, & Alexander, 2015).

At a patch scale, we found overall eastern ponderosa system responses to mixed‐severity fire matched western ponderosa system responses to mixed‐severity fire (Keyser, Lentile, Smith, & Shepperd, 2008; Stevens‐Rumann, Sieg, & Hunter, 2012). In the first decade following a mixed‐severity fire in western ponderosa systems, live trees are known to experience high mortality (and conversely lead to greater snag densities) in high‐ and moderate‐severity patches, experience attenuated mortality (and thus leave fewer snags) in low‐severity patches, and, of course, unburned patches retain high live tree densities and lower snag densities (Allen et al., 2002; Dunn & Bailey, 2016; Eskelson & Monleon, 2018). But 27‐year post‐fire, Passovoy and Fulé (2006) found ponderosa snag densities decline sharply in the decades following the fire event; our results echo these and add that snag densities were statistically indistinguishable across fire severities (i.e., they only differed between burned vs. unburned patches). Similarly, Passovoy and Fulé (2006) found a corresponding increase in coarse woody debris cover 27‐year post‐fire as snags fell, to which we add significant differences across fire severities.

Biotic community responses were consistent with post‐fire legacy patterns observed in western ponderosa pine systems at a patch level (Fornwalt & Kaufmann, 2014; Kotliar et al., 2007). Although snag densities only differed between burned versus unburned, the unique bird and woody plant communities among fire severities indicate other material legacies persist across a fire severity gradient and continue to influence biotic communities. For instance, in the same system, Keele et al. (2019) found that, when comparing multiple forest stand structural characteristics, coarse woody debris was the strongest indicator of cavity‐nesting bird community composition 27‐year post‐fire. This difference in structure may explain some disparities between our study and others in bird‐fire severity associations: for example, Hairy Woodpeckers and Western Wood Pewees were more strongly associated with higher burn severities in other studies than in ours (Fontaine & Kennedy, 2012; Smucker, Hutto, & Steele, 2005). But conversely, we show that several species exhibited similar fire severity associations both less than a decade and 27‐year post‐fire: for example, Yellow‐rumped Warbler and Cedar Waxwing strongly declined with increasing severity, House Wren and Northern Flicker were positively associated with burn severity, and Spotted Towhee and Mourning Dove showed little relationship to burn severity (Kotliar et al., 2007; Smucker et al, 2005). And while other studies on understory woody plant community responses to mixed‐severity fire also show that higher severities promoted diversity in the first decade post‐fire (Abella & Fornwalt, 2015; Crotteau, Varner, & Ritchie, 2013; Fornwalt & Kaufmann, 2014; Halofsky et al., 2011), our study is among the first to demonstrate woody plant diversity persisting in high‐severity burn patches for nearly three decades.

Moving beyond the assumption of identical starting points on pre‐disturbance landscapes (i.e., burned vs. unburned) and quantifying the influence of past material legacies on current patterns will allow scientists to more fully disentangle the effects of fire legacies on biodiversity (i.e., information legacies) and forest persistence (Johnstone et al, 2016; Peterson, 2002). Many studies (including ours) operate under the assumption of an identical starting point for all patches (Carpenter et al, 2015; Twidwell et al, 2016). This assumption can prevent understanding of the extent to which past material legacies persist to influence structure and function of a system and similarly how these legacies interact with other disturbances and landscape features that function at different scales (Turner, 2010). For instance, our study site is fragmented by human development and has experienced frequent grazing by cattle and horses for multiple decades. How the legacies of these disturbances persist and interact with the legacies of mixed‐severity fire to alter patterns in community and structure is unknown. Studies that invest in long‐term investigation of responses to disturbance with an eye for tracking changes in biotic residuals via repeated sampling (e.g., Turner, Whitby, Tinker, & Romme, 2016) can provide direct tests of prior conditions and disturbance legacies. In the absence of pre‐disturbance data, indirect methods, such as reconstructing pre‐fire overstory structure, can be used to partially assess pre‐disturbance conditions (e.g., Keyser et al., 2008; Stevens‐Rumann et al., 2012; Dunn & Bailey, 2016), but where reconstruction is not possible, such as in aging and disappearing biotic residuals (e.g., falling snags) as in our study, these methods may prove insufficient for assessing disturbance legacies.

Evidence is building in support of adopting an “ecologically informed view” of mixed‐severity fires in forest systems (Hutto et al., 2016) instead of the command‐and‐control view of the past (Holling & Meffe, 1996; Lindenmayer & Noss, 2006). Management focused on a climax or idealized ponderosa pine system state excludes unique structures, communities, and individual species such as those we detected across a full suite of burn severities (Hutto, 2008; Hutto, Conway, Saab, & Walters, 2008), and the previous negative view of high‐ and mixed‐severity fires for ponderosa pine systems has come into question in light of recent evidence that these fires did indeed play a role in historical forest structure and function (Hutto & Patterson, 2016; Parks, Miller, Nelson, & Holden, 2014). Given the persistence of these material legacies in ponderosa pine systems for (at least) 27 years, our study supplements research highlighting how mixed‐severity fire in ponderosa pine systems can foster structural and biological diversity via persistence of multi‐decadal material legacies (DellaSala et al., 2017; Hutto et al., 2016; Odion et al., 2014).

## CONFLICT OF INTEREST

None declared.

## AUTHOR CONTRIBUTIONS

CP Roberts, VM Donovan, and CL Wonkka formulated the idea and scope of the manuscript, collected and analyzed the data, and wrote the initial draft. D Twidwell, D Wedin, DG Angeler, L Powell, and CR Allen contributed to the manuscript idea and scope formation and provided funding. All authors contributed to further drafting and revisions and gave final approval for the draft submission.

## Data Availability

All data used in this article are freely available via Dryad (https://doi.org/10.5061/dryad.3sp331p).
