# Supplementary material for: Fire legacies in eastern ponderosa pine forests
Source: Ecol Evol. 2019 Jan 16;9(4):1869–79. doi: 10.1002/ece3.4879 (PMC6392404; doi:10.1002/ece3.4879)
Supplement: Supplementary file 1 [file ECE3-9-1869-s001.docx]

# SUPPLEMENTARY TABLES

**Table S1:** Multiple comparisons of mean tree density and mean coarse woody debris at Fort Robinson State Park, Nebraska, 2016 by burn severity using linear models and Tukey post hoc tests. The first column indicates which burn severities are being compared. The following columns contain the t-values and adjusted p-values for live and snag densities. The burn severity classes represent high-severity (H), moderate-severity (M), low-severity (L), and unburnt (U).

| **CWD** | | **Tree Density** | | | | **Comparison** |
| --- | --- | --- | --- | --- | --- | --- |
|  |  | **Live** | | **Dead** | |  |
| **Adj. P-value** | **t-value** | **Adj. P-value** | **t-value** | **Adj. P-value** | **t-value** |  |
| <0.001 | -4.313 | 0.001 | 2.646 | 0.532 | 1.351 | H vs. L |
| 0.436 | -1.515 | 0.597 | 0.816 | 0.856 | -0.788 | H vs. M |
| <0.001 | -6.494 | 0.001 | 5.184 | 0.001 | 6.727 | H vs. U |
| 0.035 | 2.798 | 0.029 | -1.830 | 0.215 | -1.972 | L vs. M |
| 0.141 | -2.181 | 0.001 | 2.538 | 0.001 | 5.251 | L vs. U |
| <0.001 | -4.979 | 0.001 | 4.368 | 0.001 | 7.076 | M vs. U |

**Table S2**: Understory wood plant species observed across a burn severity gradient at Fort Robinson State Park, Nebraska, 2016. The species column indicates the scientific name of each species. The burn severity classes represent (from left to right): high-severity (H), moderate-severity (M), low-severity (L), and unburnt (U). Burn severity column values show the number of sampling locations for a given burn severity class in which species were detected. The number of asterisks indicate if a species was only observed in a single severity class (*) or a single site within a single severity class (**).

| **H** | **M** | **L** | **U** | **Species** |
| --- | --- | --- | --- | --- |
| 0 | 0 | 0 | 1 | *Acer negundo*** |
| 0 | 1 | 0 | 0 | *Ericameria* sp*.*** |
| 10 | 8 | 10 | 1 | *Gutierrezia sarothrae* |
| 0 | 1 | 0 | 0 | *Juniperus communis*** |
| 2 | 0 | 0 | 2 | *Juniperus scopulorum* |
| 0 | 0 | 0 | 4 | *Mahonia repens** |
| 2 | 3 | 4 | 14 | *Pinus ponderosa* |
| 0 | 1 | 0 | 5 | *Prunus americana* |
| 7 | 2 | 4 | 10 | *Prunus virginiana* |
| 11 | 11 | 13 | 13 | *Rhus trilobata* |
| 6 | 2 | 0 | 2 | *Ribes americanum* |
| 0 | 0 | 0 | 1 | *Ribes aureum*** |
| 3 | 0 | 1 | 3 | *Ribes odoratum* |
| 0 | 1 | 0 | 1 | *Ribes oxyacanthoides* |
| 9 | 4 | 3 | 7 | *Rosa woodsii* |
| 9 | 5 | 2 | 8 | *Symphoricarpos occidentalis* |
| 12 | 9 | 10 | 12 | *Toxicodendron radicans* |
| 0 | 0 | 1 | 0 | *Ulmus americana*** |

**Table S3**: Multiple comparisons of understory woody plant and bird community compositions at Fort Robinson State Park, Nebraska, 2016 by burn severity using PERMANOVAs. The first column indicates which burn severities are being compared via PERMANOVA, the second column contains the initial F-values (from which unadjusted p-values derived), and the third column contains resultant p-values using false discovery rates to adjust for multiple comparisons. The burn severity classes represent high-severity (H), moderate-severity (M), low-severity (L), and unburnt (U).

| **Bird** | | **Understory Shrub** | | **Comparison** |
| --- | --- | --- | --- | --- |
| **Adjusted P-value** | **F-value** | **Adjusted P-value** | **F-Value** |  |
| 0.009 | 3.055 | 0.002 | 6.874 | H vs. L |
| 0.260 | 1.284 | 0.005 | 5.680 | H vs. M |
| 0.002 | 13.007 | 0.002 | 16.061 | H vs. U |
| 0.014 | 2.898 | 0.624 | 0.663 | L vs. M |
| 0.002 | 4.674 | 0.002 | 10.417 | L vs. U |
| 0.002 | 11.388 | 0.002 | 9.099 | M vs. U |

**Table S4:** Avian species observed across a burn severity gradient at Fort Robinson State Park, Nebraska, 2016. The burn severity classes represent (from left to right): high-severity (H), moderate-severity (M), low-severity (L), and unburnt (U). Burn severity column values show the number of sampling locations for a given burn severity class in which species were detected. The number of asterisks indicate if a species was only observed in a single severity class (*) or a single site within a single severity class (**).

| **Species** | **H** | **M** | **L** | **U** |
| --- | --- | --- | --- | --- |
| American Goldfinch | 0 | 2 | 6 | 8 |
| American Kestrel | 0 | 1 | 0 | 1 |
| American Robin | 0 | 1 | 1 | 0 |
| Audubon’s Warbler | 0 | 0 | 1 | 7 |
| Barn Swallow * | 0 | 0 | 0 | 2 |
| Black-beaked Magpie ** | 0 | 1 | 0 | 0 |
| Black-capped Chickadee | 0 | 1 | 1 | 5 |
| Blue-gray Gnatcatcher | 0 | 1 | 4 | 1 |
| Brown-headed Cowbird | 5 | 5 | 9 | 8 |
| Black-headed Grosbeak ** | 0 | 0 | 0 | 1 |
| Brewer’s Blackbird | 3 | 3 | 2 | 0 |
| Brown Thrasher ** | 1 | 0 | 0 | 0 |
| Bullock’s Oriole ** | 0 | 0 | 1 | 0 |
| Cassin’s Kingbird | 1 | 5 | 3 | 0 |
| Cedar Waxwing * | 0 | 0 | 0 | 3 |
| Chipping Sparrow | 0 | 0 | 3 | 3 |
| Common Nighthawk ** | 0 | 0 | 1 | 0 |
| Eastern Bluebird | 1 | 0 | 0 | 2 |
| Eastern Kingbird | 2 | 0 | 1 | 0 |
| Hairy Woodpecker | 0 | 0 | 1 | 1 |
| House Wren | 2 | 3 | 5 | 7 |
| Lark Sparrow | 2 | 7 | 0 | 0 |
| Mountain Bluebird | 1 | 0 | 2 | 1 |
| Mourning Dove | 2 | 3 | 3 | 2 |
| Northern Flicker | 5 | 2 | 3 | 0 |
| Orchard Oriole ** | 0 | 1 | 0 | 0 |
| Ovenbird ** | 0 | 0 | 0 | 1 |
| Pinyon Jay * | 0 | 0 | 2 | 0 |
| Plumbeous Vireo * | 0 | 0 | 0 | 3 |
| Prairie Falcon ** | 0 | 1 | 0 | 0 |
| Pygmy Nuthatch ** | 0 | 0 | 0 | 1 |
| Red-breasted Nuthatch ** | 0 | 0 | 0 | 1 |
| Red-headed Woodpecker | 2 | 0 | 2 | 0 |
| Rock Wren | 2 | 0 | 5 | 1 |
| Red-tailed Hawk ** | 0 | 1 | 0 | 0 |
| Spotted Towhee | 0 | 2 | 4 | 5 |
| Turkey Vulture | 0 | 0 | 1 | 1 |
| Western Kingbird ** | 0 | 0 | 0 | 1 |
| Western Meadowlark | 14 | 13 | 11 | 0 |
| Western Wood Pewee | 0 | 1 | 2 | 0 |
